# The impact of the COVID‐19 pandemic on mental health, associated factors and coping strategies in people living with HIV: a scoping review

**DOI:** 10.1002/jia2.26060

**Published:** 2023-03-13

**Authors:** Chenglin Hong, Artur Queiroz, Jordan Hoskin

**Affiliations:** ^1^ Department of Social Welfare UCLA Luskin School of Public Affairs Los Angeles California USA; ^2^ Institute for Sexual and Gender Minority Health and Wellbeing Northwestern University Chicago Illinois USA; ^3^ State of California Department of Rehabilitation Los Angeles California USA

**Keywords:** mental health, COVID‐19, people living with HIV, copy strategy, depression, scoping review

## Abstract

**Introduction:**

The COVID‐19 pandemic and associated measures implemented by authorities have created additional stressors and increased the risk of psychological illnesses among people living with HIV (PLWH). Yet, there is no collective evidence on the mental health status of this population during the global pandemic and associated factors. This scoping review aimed to synthesize the evidence in the current literature related to the mental health outcomes and challenges faced by PLWH during the COVID‐19 pandemic, identify the associated factors with psychological distress and summarize various coping strategies to ease these psychological distresses used by this population.

**Methods:**

We conducted a scoping review following the PRISMA‐ScR guideline and a literature search in four electronic databases in August 2022. Three reviewers independently screened all the search records and extracted the data from studies that met the inclusion criteria. Factors associated with worsened mental health outcomes were synthesized according to the socio‐ecological framework.

**Results:**

Among 1100 research records, 45 articles met the eligibility criteria and were included in the final review and data extraction, most of which were quantitative analyses. PLWH reported high rates of mental health problems during the pandemic. Multi‐level factors were associated with increased psychological distress, including substance use, antiretroviral adherence, social support, financial hardship and economic vulnerability during the pandemic. PLWH used social media as a coping strategy to foster social support to deal with growing mental distress. Increased mental health illnesses were associated with increased substance use, it was also found associated with suboptimal medication adherence and antiretroviral therapy (ART) care engagement.

**Discussion:**

PLWH experienced high rates of mental health illnesses, such as depression during the global COVID‐19 pandemic. There is an urgent need to provide comprehensive HIV treatment and mental health services as the pandemic continues to evolve.

**Conclusions:**

The review summarized how the mental health of PLWH was affected during the COVID‐19 pandemic. Future work in the implementation of effective interventions to promote mental health in this population is needed, not only to ensure their quality of life but also to help them maintain ART adherence and healthcare during more unprecedented times.

## 1 INTRODUCTION

The COVID‐19 pandemic continues to evolve rapidly around the globe. As of  February 2023, there are over 670 million confirmed cases globally and more than 6 million deaths caused by the disease worldwide [1]. Large‐scale government mitigation efforts, including “stay‐at‐home” orders and lockdown measures that intend to limit the number of social contacts and interactions, have slowed the exponential growth of new cases but also led to unprecedented disruptions in society, such as deepening social inequality and exacerbating health disparities among some of the vulnerable populations, such as sexual and gender minorities and people living with HIV (PLWH) [2, 3, 4]. There is growing evidence suggesting that the pandemic has already influenced the delivery of routine HIV prevention and treatment services and created barriers and challenges to the HIV prevention and care continuum [5, 6, 7]. PLWH in many settings reported having difficulty seeing their healthcare providers and refilling the antiretroviral medicines since the pandemic began [4]. These potential interruptions can lead to negative health outcomes, such as difficulties with medication adherence, increased viral load and risks of developing opportunistic infections and mental health illnesses, causing drug resistance and HIV disease progression.

In addition, the pandemic and its associated measures implemented by authorities also created additional stressors and increased the risk of psychological illnesses [8, 9]. Fear, uncertainty, loneliness, social isolation and lack of support during this unprecedented time undoubtedly contributed to worsening mental health outcomes, such as depression, anxiety, suicidal ideation and so on. This is especially concerning among PLWH, a population that already bears high burdens of poor mental health outcomes due to limited access to mental health services, internalized stigma, perceived and experienced discrimination, and other stressors [10, 11, 12]. Previous studies and reviews had consistently reported relatively high levels of depression, anxiety and other psychological disorders in PLWH compared to the general population, especially among those with other vulnerabilities, such as sexual and gender minority, people of colour, and living in low‐ and middle‐income countries [12, 13]. In fact, a cross‐sectional study found that PLWH reported significantly higher rates of depressive and anxiety symptoms than those living without HIV [14]. These mental health problems, in return, could potentially pose great threats to medication adherence and quality of life and increase the risk for negative outcomes among PLWH at each step in the HIV care continuum [15, 16].

As the global pandemic continues with the rapid spread of new variants, there is an urgent need to summarize the reported psychological distresses that PLWH continue to suffer from and provide evidence to inform the provision of HIV care services. As such, the objective of this review is to synthesize the evidence in the current literature related to the mental health outcomes and challenges faced by PLWH during the COVID‐19 pandemic, identify the associated factors with psychological distress and summarize various coping strategies to ease these psychological distresses used by this population.

## 2 METHODS

### 2.1 Search strategy

A systematic search following the Preferred Reporting Items for Systematic reviews and Meta‐Analyses extension for Scoping Reviews (PRISMA‐ScR) guidelines [17] was conducted on PubMed, Embase, PsycInfo, MEDLINE, Web of Science and CINAHL. A manual search on Google Scholar and reference lists of retrieved citations was conducted to identify additional relevant studies. We used a combination of MeSH (Medical Subject Heading) terms, keywords and phrases for the database search. We executed the database research on 4th August 2022.

### 2.2 Eligibility criteria

Studies were included if they met the following inclusion criteria: (1) assessed and reported mental health conditions as the study outcome since March 2020, given the World Health Organization characterize COVID‐19 a global pandemic on the 11th of March; (2) the study population explicitly was PLWH or in cases where the articles included both HIV‐positive participants and HIV‐negative/unknown status, we included those that provided explicit outcomes among the HIV‐positive portion; (3) utilized validated and standardized scales or tools to quantify mental health outcomes. We excluded studies that were published earlier than March 2020 or did not focus on mental health outcomes in PLWH or were not peer‐reviewed publications.

### 2.3 Study selection and screening

Following the PRISMA guideline, all the search results were imported into Covidence, a software to assist with duplicate removal and screening. First, three reviewers (CH, AQ and JH) screened the titles and abstracts of each publication independently. Any studies that appeared to meet the inclusion criteria or where the eligibility was unclear were progressed to full‐text screening. Next, the three reviewers screened full texts to determine eligibility for inclusion in the final data extraction. Results of each round were compared, and any conflicts were discussed by the three reviewers until consensuses were made.

### 2.4 Data extraction

A data extraction form was created to summarize the study characteristics and results. Data extracted from each eligible paper include title, publications year, authors, countries and regions, and participants’ characteristics. We first extracted the instruments and tools to measure mental health outcomes for mental health outcomes. Then, mental health outcomes were categorized into specific conditions, such as depression, anxiety, stress, insomnia and so on. In cases where the measurements or outcomes were not explicitly listed, we reviewed supplementary files or other sources cited by the authors. We abstracted summary measures, such as means, standard deviations and ranges, to represent the statistics on mental health outcomes. Lastly, we extracted factors associated with worse mental health outcomes and grouped them into the five‐level by the socio‐ecological model [18] and any coping strategies that were presented by the studies. Three reviewers (CH, AQ and JH) conducted and verified data extraction, and all disagreements were resolved through discussion.

## 3 RESULTS

### 3.1 Study selection

**Figure 1:**
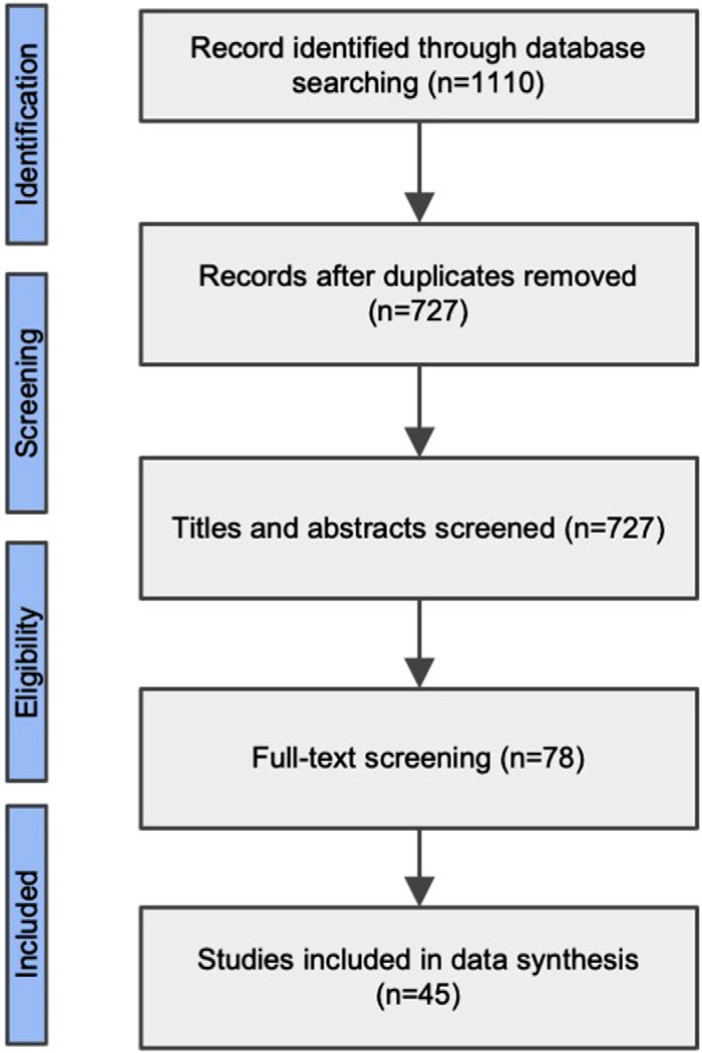
Preferred Reporting Items for Systematic Reviews and Meta‐Analyses (PRISMA) flow diagram.

From the initial systematic searches, a total of 1110 publications were identified. Of those, 283 were removed after initial screening due to duplication, and 727 articles were excluded based on screening titles and abstracts. The remaining 78 full‐text articles were assessed for eligibility, and 45 studies met the inclusion criteria and were, therefore, included in the final review and data extraction [19, 20, 21, 22, 23, 24, 25, 26, 27, 28, 29, 30, 31, 32, 33, 34, 35, 36, 37, 38, 39, 40, 41, 42, 43, 44, 45, 46, 47, 48, 49, 50, 51, 52, 53, 54, 55, 56, 57, 58, 59, 60, 61, 62, 63]. See Figure 1 for the PRISMA flowchart of the study selection and rationales for the exclusion of full‐text articles.

### 3.2 Study characteristics

Key study characteristics are summarized in Table 1. Nearly half of the studies were conducted in the United States (US, *n* = 19, 42.2%), followed by India (*n* = 3), Argentina (*n* = 2), Italy (*n* = 2), Nigeria (*n* = 2), China (*n* = 2), Vietnam (*n* = 2), Uganda (*n* = 2), Ethiopia (*n* = 1), Canada (*n* = 1), Kenya (*n* = 1), Turkey (*n* = 1), Thailand (*n* = 1), United Kingdom (*n* = 1) and Australia (*n* = 1). Two studies (4.4%) collected data from global participants. Studies were predominantly quantitative (*n* = 36, 80%), and only three (6.7%) and six (13.3%) were qualitative and mixed method, respectively.

**Table 1:** Characteristics of included studies examining mental health among people living with HIV during the COVID‐19 pandemic (*n* = 45)

	*n* (%)
*Study location* ^a^	
United States	19 (42.2%)
India	3 (6.7%)
Argentina	2 (4.4%)
Italy	2 (4.4%)
Nigeria	2 (4.4%)
China	2 (4.4%)
Vietnam	2 (4.4%)
Uganda	2 (4.4%)
Ethiopia	1 (2.2%)
Canada	1 (2.2%)
Kenya	1 (2.2%)
Turkey	1 (2.2%)
Thailand	1 (2.2%)
United Kingdom	1 (2.2%)
Australia	1 (2.2%)
Global sample	2 (4.4%)
*Study design*	
Quantitative	36 (80.0%)
Qualitative	3 (6.7%)
Mixed method	6 (13.3%)
*Study population*	
Men who have sex with men	5 (11.1%)
People who use drugs	3 (6.7%)
Older population (50 years old +)	3 (6.7%)
Youth	2 (4.4%)
Female sex worker	2 (4.4%)
Transgender individuals	1 (2.2%)
Black and Latino	1 (2.2%)
Pregnant women	1 (2.2%)
General population	26 (57.8%)

^a^ In total, more than 100% because some studies collected data in more than one country/location.

While more than half of the studies surveyed the general individuals living with HIV (*n* = 26, 57.8%), five studies (11.1%) were among men who have sex with men. Other populations that emerged included people who use drugs (*n* = 3, 6.7%), the older population (*n* = 3), youth (*n* = 2), female sex workers (*n* = 2), transgender individuals (*n* = 1) and pregnant women (*n* = 1). One study was conducted on only Black and Latino individuals. A complete data extraction record can be found in Supplementary Materials.

### 3.3 **Assessment** t**ools**

Studies used various measurement scales and tools to assess mental health outcomes (Table 2). The most commonly used tools are the Patient Health Questionnaire (PHQ, *n* = 9, 20%, including PHQ‐2, PHQ‐4, PHQ‐8 and PHQ‐9) and the General Anxiety Disorder (GAD, *n* = 9, 20%, including GAD‐2 and GAD‐7). Six studies (13.3%) used the Center for Epidemiologic Studies Depression Scale (CES‐D, including CES‐D 10 and CES‐D 20) to measure depression.

**Table 2:** Mental health outcomes and measurements among included studies

	*n* (%)
*Mental health outcomes* ^a^	
Depression	26 (57.8%)
Anxiety	21 (46.7%)
Loneliness	10 (22.2%)
Stress	8 (17.8%)
PTSD	3 (6.7%)
Sleep disorders	2 (4.4%)
Others	11 (24.4%)
*Measurements*	
GAD‐7/GAD‐2	9 (20%)
CES‐D 10/CES‐D 20	6 (13.3%)
UCLA loneliness	3 (6.7%)
PHQ‐2/PHQ‐4/PHQ‐8/PHQ‐9	9 (20%)
HADS	2 (4.4%)
Pandemic stress index	2 (4.4%)
IES‐R	2 (4.4%)
MHI‐5	1 (2.2%)
BDI‐II	1 (2.2%)
18 items PTSD	1 (2.2%)
Jenkins sleep problems scale	1 (2.2%)
Perceived stress scale	1 (2.2%)
Beck Anxiety Inventory (BAI)	1 (2.2%)
DASS‐21	1 (2.2%)
PTSD Civilian Checklist	1 (2.2%)
PC‐PTSD	1 (2.2%)
Others	3 (6.7%)
Did not specify	13

^a^ In total, more than 100% because some studies assessed more than one mental health outcome and used more than one assessment tool.

The UCLA Loneliness Scale was frequently used (*n* = 3) in studies measured loneliness among their participants. Post‐traumatic stress disorder (PTSD) was measured by the Primary Care PTSD Screen and the Post‐Traumatic Stress Disorder Checklist for Civilians. Stress was assessed using the Pandemic stress index and the Perceived stress scale. Two studies used the Impact of Event Scale to assess the participants’ feel in response to the COVID‐19 pandemic. Other assessment tools utilized were Hospital Anxiety and Depression Scale (HADS), the Mental Health Inventory, the Beck Depression Inventory (BDI‐II), the Beck Anxiety Inventory (BAI), the Jenkins Sleep Scale (JSS) and the Depression, Anxiety and Stress Scale (DASS‐21). Three studies used other assessment tools, and 13 (28.9%) did not specify which tool was used.

### 3.4 Mental health outcomes

Twenty‐six (57.8%) studies assessed depression or depressive symptoms (Table 2) [19, 20, 22, 23, 26, 29, 31, 32, 34, 35, 36, 38, 40, 41, 42, 44, 45, 48, 49, 50, 51, 53, 54, 57, 58, 62]. While most of the studies assessed depression or depressive symptoms cross‐sectionally in a specific recall period, a few studies asked if the participants were experiencing more severe symptoms than usual and assessed the longitudinal changes. For example, Pantelic et al. found that 71.8% of the participants reported feeling more depressed than usual [48]. In a study in which researchers conducted a month‐12 follow‐up assessment, the rates of depression symptoms nearly tripled from month 12 to the post‐lockdown assessment [57] Similarly, 21 studies (46.7%) assessed anxiety [19, 22, 25, 26, 31, 32, 33, 35, 36, 38, 39, 40, 41, 43, 48, 50, 51, 54, 56, 59, 62] and 11 studies assessed loneliness [21, 23, 26, 27, 31, 32, 37, 42, 44, 46, 62]. The stress of dealing with the pandemic was assessed in eight studies [21, 23, 32, 37, 42, 44, 46, 62], and other mental health outcomes examined in these studies include schizophrenia, attention deficit hyperactivity disorder, autism, intellectual delay, insomnia, bipolar disorder, panic disorder and PTSD [20, 22, 28, 30, 33, 37, 38, 40, 55, 60, 61, 64].

Worsening mental health outcomes were also identified in qualitative studies. For example, participants in Pantelic et al.’s studies said,

*“Just got very anxious… Could not sleep some nights”* [48]

Another qualitative study conducted in Thailand also revealed the fears of COVID‐19 and concerns about their health among a group of men who have sex with men (MSM) living with HIV,

“I experienced extreme anxiety and fear during COVID‐19 mostly due to personal health reasons and since I have low immunity. A colleague at my office got infected with COVID‐19, which made me even more concerned.” [47]

These psychosocial issues may further create a syndemic condition exacerbating adverse health outcomes among PLHIV.

### 3.5 Factors associated with negative mental health outcomes

**Figure 2:**
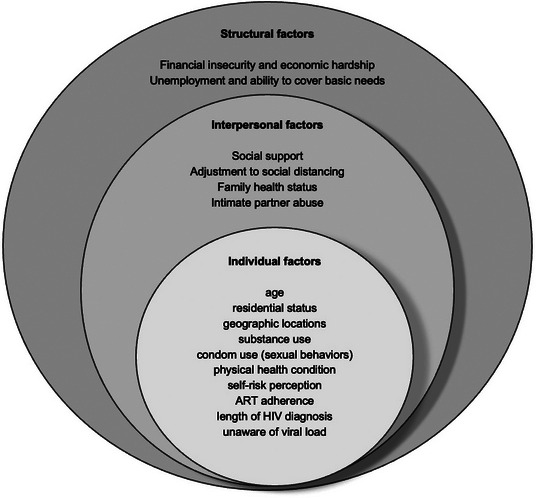
Factors associated with mental health outcomes among people living with HIV during the COVID‐19 pandemic.

Negative mental health outcomes were found to be associated with multi‐level factors in included studies. We structured these factors according to the different levels of the socio‐ecological framework. As shown in Figure 2, individual, interpersonal and structural factors were the most reported in the included studies.

At the individual level, age and gender were significant predictors of mental health outcomes. Several studies found that older PLWH were more likely to experience psychological distress during the pandemic [20, 32, 34]. For example, Abate et al. found that pregnant mothers in Ethiopia aged 30 and above were more likely to experience depression than those under 30 years old [20]. In Italy, researchers also found that PLWH who were older than 60 were more likely to be screened for psychological outcomes [32]. However, the findings were not consistent. One study among older adults living with HIV suggested that those who were aged 50–63 years had higher PTSD severity scores than those who were 64 years old and above [46]. Two studies discovered that female individuals living with HIV were more likely to experience mental health distress. There was also evidence that mental health outcomes were associated with residential status and geographic locations. For example, Abate et al. found that PLWH being urban residents were nearly twice as likely to be depressed compared to those being rural [20], and in another study in two Chinese municipalities, individuals who were non‐local residents were experiencing more mental health problems [39]. Jones et al. discovered that participants in the United States reported higher levels of depressive symptoms compared to those from Argentina [42]. In a web‐based cross‐sectional study, Siewe Fodjo et al. found that Eastern European residents experienced higher rates of anxiety than those in Western Europe (30.5% vs. 17.5%, *p* = 0.062) [19]. In their follow‐up survey, Siewe Fodjo et al. also found that PLWH in Latin American countries reported the highest rates of anxiety and depression compared to those in Eastern and Western Europe [51]. Multiple studies also illustrated the positive relationships between substance use and mental health outcomes, including binge drinking, marijuana and cocaine use, and substance misuse [24, 31, 33]. In addition, one study also suggested that those who had more frequent condom use during sex and had either increased or decreased numbers of sexual partners experienced more general distress after the COVID‐19 outbreak [44].

Other frequently reported factors associated with mental health outcomes were one's physical health condition and self‐risk perception. It was suggested that those who had previous mental health issues, such as psychological disorders and a lifetime history of mood disorders, were more likely to experience anxiety and depressive symptoms [50, 56]. Besides, studies also found that mental health issues were related to poor antiretroviral adherence, having a few years of HIV diagnosis and not being aware of HIV viral load [29, 32]. In addition, PLWH reported higher rates of self‐risk of catching COVID‐19. Some were unsure about the presence of individuals with COVID‐19 around them and were concerned that they were taking insufficient precautions to protect themselves [43, 56]. Others also worried about their weakened immune status [47, 48, 49]. These concerns and worries may explain why individuals who required more information on COVID‐19 prevention were at higher risk of anxiety [32]. Lastly, the fear of HIV status disclosure and stigmatization also created additional stressors [47, 61].

At the interpersonal level, mental health outcomes were associated with social support and changes in interpersonal relationships in accordance with COVID‐19‐related measures. Studies found that lower levels of social support were correlated with higher depressive and anxiety symptoms [42, 43, 44]. PLWH also felt lonely and had no one to confide in due to increased social isolation [28, 49, 52], which are their memories of the early days of HIV [48, 52]. PLWH reported that the most stressful things were adjusting to social distancing [21], and some also cited a lack of trust in their community members for following the COVID‐19 guideline [49]. Other determinants of mental health outcomes include family health status [30] and in a study among female sex workers living with HIV, increased emotional partner abuse was marginally significantly associated with COVID‐19‐related mental health challenges [59]. Having public‐facing jobs during the pandemic also created additional stressors that caused constant worry leading to worsened mental health [49].

At the structural level, financial insecurity and economic hardship were also associated with mental health outcomes during the pandemic [21, 23, 61]. Unemployment [25, 38, 44], working less during the pandemic [28], not having enough money/income [28, 38, 49, 53], ability to cover basic needs [23] and lower education level [49] were found to be correlated with various mental health distresses in included studies. Indeed, one study found that those with stable salaried jobs or who are self‐employed had less mental health distress during the pandemic [30]. Qualitative research also provided evidence to support these insights,

“I stay alone. I used to run a beauty salon that I rented, which is now closed. I have no money to pay the owner who is asking for rent. I have no savings and no one to talk to. I have a lot of tension and I feel lonely.” [43]

“Currently, we have the COVID pandemic, I do not have the money and most of the businesses were shut down. Where I used to spend about ten thousand Uganda shillings for my transport, I am now spending about forty thousand Uganda shillings. Therefore, in all these ways I must experience worry or apprehension” [61]

### 3.6 Mental health as a predictor of other health‐related outco**mes**

Several studies also considered mental health outcomes as predictors and examined their associations with other health‐related outcomes, including substance use and treatment adherence. In a prospective cohort of PLWH, Meanley et al. found that individuals with higher depressive symptoms were more likely to report substance use outcomes, including binge drinking, daily marijuana use and recreational drug use [45]. Two other studies by Marbaniang et al. and Ekstrand et al. showed that a worse mental health outcome, specifically general anxiety and depression, can lead to suboptimal ART adherence and engagement in ART care [29, 35, 43]. Similarly, in a study conducted in western Kenya, Enane et al. found that suffering from mental health issues hinders adolescents’ connections and engagement with health services [36].

### 3.7 Coping strategies

Despite being limited, some analyses are relevant to understanding the general process of how COVID impacted mental health status and how individuals cope with these distresses. Gwadz et al. brought a wide range of coping strategies employed to deal with the emotional effects of COVID during the pandemic initial times, especially creating new routines that include accessing social support networks, exercises, meditation, art, spirituality, prayer and interactions with family and friends. These last ones seem to have increased during this time, but mainly via telephone, smartphone and social media [38]. The use of social media for social support was further explored in a qualitative study that reported on how these tools were used to reach out to family and friends and strengthen social connections, in search of emotional connections, and to re‐establish a semi‐normal routine [52]. Another study also discovered the effectiveness of an online mindfulness lesson in reducing feelings of depression, anxiety and loneliness among older adults living with HIV [27].

## 4 DISCUSSION

This review aimed to identify the effects of the COVID‐19 pandemic on the mental health of PLWH worldwide. Our review of 45 articles found multi‐level factors associated with mental health outcomes, mainly focused on the screening of depression, anxiety and stress. Substance use, loss of social and economic support, and difficulties in accessing health services were some of the main associated factors, as well as reflected in the coping strategies which seek to mitigate or relieve them. These disruptions in treatment, which already had been worsened by the COVID pandemic itself, can severely impact the HIV care cascade, increasing the number of new infections, increasing viral load in previously undetectable individuals and worsening the overall health of PLWH. Of note, most of the studies were cross‐sectional and quantitative analyses. Future research should consider collecting longitudinal data and utilizing qualitative approach to better investigate the impact of the pandemic on mental health outcomes among this population.

The application of the syndemic approach to health research has rapidly increased with the emergence of COVID‐19. Added to the other current pandemic, such as HIV, COVID‐19 may disproportionately impact communities already facing longstanding inequities [65]. In a recent review addressing these syndemic aspects, the authors showed that most of the studies that assessed mental health research focused on linkages between mental health and/or psychosocial challenges and sexual practices that elevate HIV exposure or reduce retention in HIV care [66]. The new reality of having to manage two infectious diseases simultaneously has brought a new level of stress to PLWH, especially those dealing with stigma closely. PLWH who concealed their HIV status due to fear of stigma and discrimination face the threat of unwanted exposure of their HIV status to family members due to the mailing of medicine and interpersonal proximity during this time. A survey in China indicated that medication uptake and HIV care disruption, risk of status exposure and heightened HIV stigma may have contributed to worsened mental health among PLWH during the COVID‐19 outbreak [54]. The combined effects of the syndemics with poor mental health can lead to suboptimal medication adherence, failure to achieve viral suppression and HIV transmission risk.

This poor mental health state is likely associated with periods of physical distancing restrictions. PLWH, particularly older PLWH, are often in a situation of forced social isolation (mainly due to stigma). Previous social or cultural coping mechanisms developed throughout the years by these people may have been destroyed due to social distancing. This situation creates a dilemma in which PLWH may see themselves facing the desire to defy social distancing policies and meet coping mechanisms, causing them to develop additional stress due to fears of SARS‐CoV‐2 exposure or stigma [67]. Diminished neurocognitive functioning and heightened mental health burden evidence in older PLWH may impede effective self‐care [68]. PLWH have an increased likelihood of mental health burden, illicit drug use and other sexually transmitted infections, all catalysed by psychosocial burdens experienced at elevated rates in marginalized populations, including sexual and gender minorities, racial and ethnic minorities, and/or the poor and underserved.

While studies illustrated the complex and multi‐level needs of PLWH reported on the growing need to support their physical and mental health during the COVID‐19 pandemic, they reported difficulties accessing essential health services and information on how to take care of themselves during this pandemic [69]. Another study that analysed coping strategies and resilience in PLWH compared to people living with oncological diseases during the pandemic reports that worse mental health outcomes can affect their resilience, reinforcing the importance of the prevention and management of mental health in this population [63]. In this study, Folayan et al. point out that PLWH was significantly associated with lower odds of reporting post‐traumatic stress symptoms. We hypothesize that this could be the result of a built‐in resilience of having to be part of a previous and ongoing pandemic.

New strategies to manage this decline in mental health also ought to be created, adapted or implemented. For example, one HIV clinic in Chicago, Illinois, reported that some patients who had been receiving mental health counselling before social distancing measures temporarily discontinued services when they were offered via telehealth, but other patients engaged in telecounselling for the first time [4]. Engagement in telecounselling was universal among patients with stable income and housing but entirely absent among patients who were unstably housed with no steady source of income; in lieu of telecounselling, the latter group of patients received peer counselling, which was more flexible with respect to the time and locations in which it could occur. This is just one of the examples that illustrate how the pandemic has affected aspects of health equity, especially from a social and economic perspective in places with an unstable or challenging economy. This evidence demonstrates how economic and social aspects are indivisible from mental health, which in turn cannot be treated completely separately from the broad concept of health. Addressing these concepts individually may often be necessary for logistical or argumentative reasons, but their effects are transversal. Macro‐policies that guarantee the security and financial stability of individuals should not be ignored or prioritized over their psychological or physical needs.

Our review has some limitations. We focused on thematically assessing the studies, but we did not measure the rigour of the studies. Secondly, due to the heterogeneity of included studies, a meta‐analysis was not conducted to derive aggregate statistical evidence on the prevalence of mental health outcomes among PLWH. Lastly, we only selected published studies in English. It is possible that studies in other languages and those in the grey literature were missed.

## 5 CONCLUSIONS

The review summarized how the mental health of PLWH was affected during the COVID‐19 pandemic, showing trends of worsening of previous conditions or development of new symptoms. Although the studies were collected and focused on the unique perspective and experience of individuals, many articles point out how these are reflections of external actions and beyond the control of these individuals, such as infections, deaths and government responses. Mental healthcare during the pandemic, a serious health issue in itself, becomes even more serious when we demonstrate that there is evidence that this deterioration in mental health can be reflected in a worsening of existing clinical conditions, such as an unsuppressed viral load. Future work in the implementation of effective interventions to promote mental health in this population is needed, not only to ensure their quality of life but also to help them maintain ART adherence and healthcare during more unprecedented times. These interventions should not only focus on individual coping mechanisms but should also address the social and structural barriers they face as individuals living with HIV.

## AUTHORS’ CONTRIBUTIONS

CH conceptualized, designed, initiated the study and conducted the initial search. CH, AQ and JH completed article selection, screening, review and data collection. All authors assisted with the interpretation of the results. CH and AQ drafted the manuscript. All authors critically reviewed and commented on the drafts and approved the final version of the manuscript.

## COMPETING INTERESTS

All the authors declare no competing interests.

## Supporting information


**Table S1**: Characteristics and key findings of study included in this review

## Data Availability

All study data were from previously published studies.
