# Supplementary material for: The impact of the COVID‐19 pandemic on mental health, associated factors and coping strategies in people living with HIV: a scoping review
Source: J Int AIDS Soc. 2023 Mar 13;26(3):e26060. doi: 10.1002/jia2.26060 (PMC10009802; doi:10.1002/jia2.26060)
Supplement: Supplementary file 1 — Table S1: Characteristics and key findings of study included in this review [file JIA2-26-e26060-s001.docx]

**Supplement table: Characteristics and key findings of study included in this review**

| **First author (year)** | **country/setting** | **study design** | **study population characteristics** | **sample size** | **mental health outcomes** | **Assessment tools** | **prevalence of reported outcome (depression, anxiety, stress, insomnia)** | **associated factors** | **coping strategies** |
| --- | --- | --- | --- | --- | --- | --- | --- | --- | --- |
| Abate (2021) | Ethiopia | Quantitative | HIV-Positive Pregnant Women | 291 | Family History of psychiatrich problems and Depression | PHQ-9 | Depression: 28.7% [95% CI (24.3–33.4)] | (1) being ≥30 (2) being an urban residency  (3) Having a first pregnancy <18 years  (4) having known HIV serostatus during pregnancy. | n/a |
| Algarin (2020) | United States | Quantitative | n/a | 16 | COVID-19 stress | n/a | The average level of stress reported by our sample due to COVID-19 was 4.4 ± 3.3 with scores ranging from 1 to 10 | Among the most stressful things participants reported were potential exposures to SARS-CoV-2, adjust- ing to social distancing, and issues related to finances. One participant reported a perceived benefit, noting that the connection to an emergent social support system had decreased their perceived loneliness. | n/a |
| Armbruster (2020) | United States | Quantitative | youth with HIV | 76 | Depression Anxiety Bipolar disorder Schizophrenia ADHD Autism Intellectual delay | n/a | Depression: 32% Anxiety: 5% depression and anxiety: 21% Bipolar disorder: 3% Schizophrenia: 1% ADHD: 4% Autism: 1% Intellectual delay: 3% | n/a | Telemedicine options  (e.g., 22-year- old female patient with busy work schedule & increased anxiety due to COVID-19 was able to schedule an evening TM mental health visit which improved her symptoms) |
| Ballivian (2020) | Argentina | Quantitative | n/a | 1336 | depression perceived stress loneliness | CES-D 10; Perceived stress scale; UCLA loneiness scale | 0 | economic hardship | Individuals endorsing more resilience, the relationship between economic hardship and the outcomes of overall mental health, perceived stress, and loneliness was buffered by resilience |
| Baum (2022) | United States | Quantitative | n/a | 1430 | anxeity | GAD-7 | n/a | PLWH had lower odds of anxiety (OR=0.67, 95% CI: 0.51–0.89) and higher odds of high resilience (OR=1.21, 95% CI: 1.02–1.44) than HIV-uninfected participants, adjusted for covariates. | The presence of anxiety was associated with higher risk of misuse of all substances. High resilience was associated with lower risk of anxiety and misuse of sub- stances, adjusted for covariates. |
| Beer (2021) | United States | Quantitative | n/a | 0 | generalized anxiety disorder (GAD) | n/a | People living in households with incomes >=400% of FPL were the only group where the observed prevalence of depression and anxiety symptoms during the COVID-19 period was higher ; in this group, the prevalence of depression symptoms was 9% compared with a predicted value of 5% and the prevalence of anxiety symptoms was 11% | Authors found elevated levels of symptoms consistent with major or other depression and generalized anxiety disorder among PWH living in households with higher incomes. This group had the lowest predicted estimate for unemployment, and as such may have experienced increased mental health issues when confronted with unexpected lack of job stability. | n/a |
| Berko (2021)* | United States | Quantitative | n/a | 214 | Depression Anxiety Loneliness | CES-D-10 GAD-7 Three-item Loneliness Scale (3IL) | n/a | n/a | online mindfulness lessons produced significant reductions in depression, anxiety, and daily loneliness. |
| Berko (2021)* | United States | Quantitative | older people living with HIV (age 50+) | 671 | Loneliness | Three-item Loneliness Scale (3IL) | n/a | Black participants were less lonely than White participants (5.2 vs 5.6, P=.02). In the multivariable linear regression analysis, Black participants were again less lonely than Whites (coef. -0.45, P=0.01) while controlling for age, education, depression, anxiety, number of co-morbid conditions, being single, income, gender, sexual orientation, and spirituality (Adjusted R2=0.38; P<.01). | n/a |
| Brouillette (2021) | Canada | Quantitative | People Aging with HIV | 77 | psychological distress | HADS | 39.5% of the participants experienced an increase in psychological distress between the pre-COVID-19 and the first wave of the COVID-19 period, and 32.5% met the definition of psychological distress in the clinical range. | feeling lonely quite often or not having someone to confide in was associated with distress, as was not feeling rested in the morning. Work loss because of COVID-19, not having enough money to meet one’s needs, or worrying about such a possibility was predictive of distress | n/a |
| Campbell (2022) | South Africa | Quantitative | n/a | 83 | depression | n/a | over a quarter of respondents stated they felt more depressed since the arrival of COVID-19 (29%), and since the start of the lockdown (27%) | depression was associated with poorer ART adherence | n/a |
| Chindarkar (2021) | India | Quantitative | n/a | 647 | psychological well-being | MHI-5 | In terms of psychological well-being status, the mean MHI-5 index is 16.858 (SD = 172) | family health status is statistically significantly correlated in the expected direction with MHI-5 and conflict in personal relationships. those in salaried jobs and self-employment have a less positive mental state compared to those in daily wage work. job insecurity and financial insecurity have a negative and statistically significant association with MHI-5. | n/a |
| Cooley (2021) | United States | Quantitative | n/a | 135 | Depression Anxiety Loneliness | BDI-II HADS UCLA Loneliness Scale | Beck Depression Inventory—II (BDI-II) Score - 13.8 (11/4) Change in BDI-II Score since previous study visit; + 5.0 (6.7)  Hospital Anxiety and Depression Scale (HADS)—Anxiety Subscale Score - 7.7 (4.4) Change in HADS- Anxiety subscale score since previous visit - +2.1 (2.3)  UCLA Loneliness Scale- 23.0 (13.9) | Greater marijuana use was significantly correlated with higher symptoms of depression (r = 0.25, p = 0.008) and anxiety (r = 0.22, p = 0.01) | n/a |
| Delle Donne (2021) | Italy | Quantitative | n/a | 98 | Depression Stress Anxiety | IES-R DASS-21 | 45% revealed from mild to severe psychological impact from COVID-19 according to IES-R  Depression (14%), anxiety (11%), and stress (6%) according to DASS- 21. | Requiring further information on prevention of COVID-19 infection was associated to a higher risk for mild-to-severe levels of anxiety (OR 5.06; 95% CI 1.25/20.51; p=0.023) measured by DASS-21.  female gender, age, fewer years from HIV diagnosis and not being aware of their own viremia were associated to a higher risk of negative psychological outcomes.  Women, elderly patients and those with recent HIV diagnosis appear to be the more psychologically fragile subgroups | n/a |
| Diaz-Martinez (2021) | United States | Quantitative | n/a | 116 | Anxiety COVID-19-related worry | GAD-7 COVID-19-related worry was assessed by asking partici- pants “On a scale of 1 to 10, how worried are you about the COVID-19 pandemic? 1 being not worried at all and 10 being extremely worried.” Scores of ≥ 6 were considered high levels of worry. | Anxiety symptoms (12.9%) COVID-19-related worry (64.7%) | Compared to HIV-uninfected par- ticipants, PLWH had lower odds of having anxiety symp- toms (OR = 0.39, 95% CI 0.18–0.81; χ2 = 6.31, P = 0.012) and lower odds of high levels of COVID-19-related worry (OR = 0.42, 95% CI 0.21–0.83; χ2 = 6.21, P = 0.013), as well as twofold higher odds of high resilience (OR = 2.01 95% CI 1.13–3.60; χ2 = 5.59, P = 0.018). | n/a |
| Dyer (2020) | Kenya | Quantitative | n/a | 486 | depression | PHQ-9 | 9% (n = 45) with mild depression symptoms, and 1% (n = 3) with moderate-to-severe depression symp- toms | Young adults 20–24 years old had more mild to severe depressive symptoms than the younger age groups (p < 0.001). | n/a |
| Ekstrand (2022) | India | Quantitative | n/a | 467 | anxiety depression | n/a | anxiety (16.5%)  depression (6.4%) | n/a | n/a |
| Enane (2021) | Kenya | Quantitative | adolescents retained in or recently disengaged from HIV care | 334 | anxiety depression | PHQ-2 GAD-2 | Adolescent scores on the PHQ-2 were categorized as 0–2 (94.4%) vs. 3 or higher (5.6%), with those 3 or higher meeting the threshold for further evaluations for possible depression.  Similarly, scores on the GAD-2 ranged from 0–2 (94.8%) to 3 or higher (5.2%), with those 3 or higher meeting the threshold for further evaluations for possible anxiety. | n/a | n/a |
| Focà (2022) | Italy | Quantitative | n/a | 315 | stress and experiences | n/a | worried: (47.9%) anxious: 26 (15.6%) isolated: 22 (13.2%) | n/a | n/a |
| Folayan (2022) | Nigeria | Quantitative | n/a | 2965 | PTSD | 17-item self-report questionnaire that measured PTSS | PTSD: 1245 (42.0) | n/a | n/a |
| Gwadz (2021) | United States | mixed-methods | Black and Latino living with HIV | 122 | worry sleep anxiety depression | Pandemic Stress Index | changes to the normal sleep pattern: 78.1% More anxiety: 69.8% loneliness: 66.7% More depression: 56.2% | Participants reported feeling confused, anxious, and “paranoid,” and noted heightened concerns about their risk for contracting COVID-19 related to comorbidities common in this sample and among their loved ones such as chronic obstructive pulmonary disease, diabetes, and cancer, in addition to HIV. Participants also expressed anxiety related to their inability to avoid densely populated areas. They described the added burdens of caring for and some-times even losing individuals close to them to COVID-19. Some participants noted that their concerns about con- tracting COVID-19 were so great that they voluntarily admitted themselves into the hospital for health conditions they mistook as COVID-19 | A wide range of strategies to cope with the emotional effects of COVID were identified including accessing social support networks, medlitation, art, spirituality, prayer. Access to phones and internet was critical.Several participants spoke about creating everyday routines and strategies to manage COVID-19 related anxieties. For example, they described the different ways they tried to keep themselves distracted. These included hobbies, exercise and other ways of staying physically active, and calming and entertaining activities that could be done indoors, including prayer and meditation. Interactions with family, friends, and support networks, where possible, were also important ways of easing COVID- 19-related anxieties. Participants also described that engagement with family and friends increased compared to the time before COVID- 19, but mainly via telephone, smartphone, and social media. Participants also created substi- tutes for social interaction that could provide some form of contact with people while keeping them safe using substances, albeit not necessarily at a level that was heavy or hazardous, was a way of dealing with loneliness and boredom due to COVID-19 restrictions. “Keeping busy” was a common theme that included examples such as moving “hangouts” and conversations with friends online;reading, watching television and movies; journaling; getting to know one’s neighbors; learning to cook; trying new reci- pes; exercising (including exercising in the house); engaging in religious or spiritual practices such as prayer or medita- tion; volunteering at food pantries; helping neighbors; pro- viding health information to others; starting arts and crafts projects; initiating online education classes; and handling substance use issues. |
| He (2022) | China | Quantitative | MSM | 943 | sleep anxiety | Jenkins sleep problems scale  GAD-7 | 44% and 20% of the participants experienced insomnia symptoms and moderate/severe GAD, respectively. About 6% had ever sought professional psychological consultation and support due to mental pressure | In multivariate analysis, mental health problems was positively associated with non-local residents and HIV-related healthcare interruptions during the COVID-19 pandemic. | n/a |
| Hochstatter (2020) | United States | Quantitative | people living with HIV and substance use disorder | 64 | Anxiety/social anxiety bipolar/manic depression  depression panic disorder PTSD other | N/A | Anxiety/social anxiety=19(30%) bipolar/manic depression=11(17%) depression=33 (52%) panic disorder=1(2%) PTSD=5(8%) other=7(11%) | n/a | n/a |
| Javanbakht (2022) | United States | Quantitative | MSM | 181 | depressive symptomatology anxiety | CES-D 20 GAD-7 | CES-D 20 score: 16 moderate to severe anxiety: 31.1% | n/a | n/a |
| Jones (2021) | United States Argentina | Quantitative | n/a | 1554 | Depression symptomatology Perceived stress loneliness | CES-D 10 Percevied Stress Scale UCLA loneliness scale | main score: Depression: 12.84 (4.56) Stress: 8.27 (1.16) Loneliness: 3.74 (3.77) | n/a | n/a |
| KUMAN TUNÇEL 2020 | Turkey | Quantitative | n/a | 307 | anxiety | Beck Anxiety Inventory (BAI) | 79 (25.7%) of the participants were defined as having anxiety, as the result of having a BAI score higher than 16. | As a result, having anxiety was found to be significantly associated with having a psychiatric disorder (P = 0.002, OR = 3.02, 95% confidence interval (CI) = 1.49–6.13), the perception of taking insufficient precautions to protect oneself from COVID-19 (P = 0.002, OR = 2.75, 95% CI = 1.47–5.16), being unsure about the presence of an individual with COVID-19 near oneself (P = 0.023, OR = 1.94, 95% CI = 1.09–3.44), and living with a household member with a chronic disease (P = 0.022, OR = 1.92, 95% CI = 1.10– 3.37). | n/a |
| Marbaniang (2020) | India | mixed-methods | n/a | 167 | anxiety | GAD-7 | prevalence of generalized anxiety was 25% (n = 41) | The following four themes were identified in thematic analysis: a) concerns related to the immediate present; b) concerns related to the imminent future; c) lack of social and financial support; and d) indifference to circumstances secondary to COVID-19  PLHIV with GAD-7 score ≥ 10 had fewer remaining doses of ART than those with lower GAD-7 scores (p = 0.05) | n/a |
| Matsumoto (2021) | Vietnam | Quantitative | n/a | 1243 | depression, anxiety, and stress. | DASS-21 | The mean total DASS-21-V score was 9.2 in this population, with a 7.6% prevalence of general distress | Lack of social support, lost job, changed jobs, received ART in another hospital. | n/a |
| Meanley (2022) | United States | Quantitative | n/a | 2121 | Depression | CES-D 10 | 40% reported significant depressive symptoms. | depression was associated with binge drinking, daily marijuana use, and recreational drug use | n/a |
| Nguyen (2021) | United States | Quantitative | Older adults living with HIv | 100 | Stress | Pandemic Stress Index, PTSD Civilian Checklist | Almost half (54.0%) experienced changes in sleep patterns Many respondents reported feeling anxious (56.0%), frustrated (50.0%), depressed (41%), and bored (43.0%). On the social isolation scale, the mean response was 6.7 (SD = 2.7) and 48% reported a score of 8 or higher. Respondents had a mean PTSD sum score of 34.2 (SD = 13.3) with a range from 10 to 80. | higher PTSD severity score (OR = 1.09, CI [1.04, 1.15]; p = .001) and inability to attend a provider appointment (OR = 4.56, CI [1.29, 16.07]; p = .02) were significantly associated with greater odds of missing a dose | n/a |
| Nitpolprasert (2021) | Thailand | Qualitative | MSM | 26 | n/a | n/a | The interview data revealed three primary themes, including: (1) anxiety related to COVID-19, including fear of contracting COVID-19 and concern about HIV status disclosure, (2) anticipated and experienced impact of COVID-19 on employment, financial health and relocation, and (3) impact of COVID-19 on HIV service delivery and ART refill and clinic visit retention. | n/a | n/a |
| Pantelic (2021) | United Kingdom | mixed-methods | n/a | 653 | Anxiety  Depression | n/a | 501 (77.6%) respondents reported feeling more anxious than usual; 464 (71.8%) reported feeling more depressed than usual; 128 (19.8%) reported having suicidal thoughts; 472 (73.1%) reported having difficulties sleeping. | Worsening mental health was a common theme identified throughout the qualitative analysis, with many of the responses indicating specific concerns around increased anxiety and depression: “My mental health has deteriorated quite dramatically. [The pan- demic] has made me more anxious and hypersensitive”. Perceptions of increased vulnerability to Covid-19 due to HIV also seemed to affect mental health.  In some cases, the Covid-19 pandemic triggered memories of the early days of HIV | n/a |
| Parisi (2021) | United States | mixed-methods | n/a | 227 | Depression | PHQ-8 | Sixty-nine participants (30.4%) indicated their men- tal health worsened due to the pandemic compared with 19 (8.4%) who indicated improvement and 139 (61.2%) who reported no change. Beyond baseline depression measured by PHQ-8 score (p = 0.004) and education level (p = 0.020), there were no statistically significant differ- ences in changes in mental health by baseline character- istics. Over half of those with moderate depression expe- rienced worsened mental health compared with 42.9% of those with severe depression, 28.8% of those with mild depression, and 16.5% of those with minimal or no depres- sion. | Having more than a high school education was associated with increased odds of reporting worsened mental health (odds ratio: 3.47, 95% confidence interval 1.52–7.90, χ2: 8.77, p = 0.003), rather than no change in mental health | n/a |
| Pizzirusso (2021) | United States | Quantitative | n/a | 49 | Depression, Anxiety | PHQ-2, GAD-2 | Neuropsychiatric characteristics: Mean global score 44.4 (10.9); Lifetime history of mood disorder: 78%; Active mood disorder 14%; Lifetime anxiety disorder: 61%; Active anxiety disorder 14%; Lifetime PTSD 39%; Active PTSD 6%; Symptoms of apathy 33%.  In the follow up: Twenty-one people (43%) endorsed symptoms on the GAD-2, although only 7 (14%) met threshold for anxiety disorder. | Latinx and Caucasians were also more likely to endorse any symptoms on the GAD-2;Scores on the GAD-2 were higher with lifetime histories of mood disorders; significant differences between Latinx and African Americans, Caucasians and African Americans, but not Caucasians and Latinx. | n/a |
| Rhodes (2020) | United States | Qualitative | MSM | 1554 | n/a | n/a | Participants reported that their mental health was profoundly affected by COVID-19 and the necessary precautions required to reduce risks of exposure. Feelings of Isolation, Hopelessness, and Worry are Common | Workplace Exposure Is a Worry  “I do feel alone, and it kind of reminds me of when I learned that I had HIV.”  Participants reported that their mental health was profoundly affected by COVID-19 and the necessary precautions required to reduce risks of exposure. Specially the isolation measures to avoid covid, the lack of political actions and the constant changes in the world. Another participant reported, “I do feel alone, and it kind of reminds me of when I learned that I had HIV.” | Use of Social Media for Socialization and Support Has Increased |
| Salako (2022) | Nigeria | Quantitative | n/a | 338 | Fear anxeity depression | n/a | Fear: 47.4% anxeity: 13.1% depression: 15.7% | there was a significant association between the income status and psychological challenges (OR; 1.8, CI: 1.0-3.5, P = 0.05). | n/a |
| Siewe Fodjo (2020) | 32 countries | Quantitative | n/a | 317 | Anxiety and Depression | GAD-2 and PHQ-2 | Anxiety and depressive disorders were found in 72 (22.7%) and 74 (23.3%) participants, respectively, (Table 1); 50 PLWH (15.8%) had both anxiety and depressive disorders. | anxiety tended to be more frequent among Eastern Europe residents | the unequal burden of psychosocial problems in these 2 settings could be related to a higher perceived social judgment and stigma vis-à-vis PLWH in developing countries. |
| Siewe Fodjo (2021) | Global | Quantitative | n/a | 247 | depression and anxiety | PHQ-4 | Anxiety and depressive disorders were found in 72 (22.7%) and 74 (23.3%) participants; 50 PLWH (15.8%) had both anxiety and depressive disorder | Compared to males, female PLWH showed a higher prevalence of anxiety (37.7% vs. 23.6%; p = 0.048) and depression, although not statistically significant (37.7% vs. 24.7%; p = 0.074). Regional disparities in the burden of psychosocial problems wereobserved, with PLWH in Latin American countries reporting the highest prevalence of both anxiety and depression (38.8% and 40.0% respectively), followed by Eastern European countries (anxiety: 34.1%% and depression: 34.1%%) and Western Europe (anxiety: 15.5% and depression: 16.4%); p = 0.001. Comparing the four most represented countries (Belgium, Brazil, France, Russia), the prevalence of both anxiety and depression were highest in Brazil (respectively 39.8% and 41.0%), followed by Russia (respectively 32.1% and 25.0%), then Belgium (respectively 17.1% and 19.5%) and France (respectively 14.3% and 7.1%) | n/a |
| Sun (2020) | China | Quantitative | Adult population living with HIV | 703 | depression anxiety insomnia | n/a | 60.8% reported depression, 49.8% noted anxiety symptoms, and 38.5% reported recent insomnia. | n/a | n/a |
| Tran (2022) | Vietnam | Mixed-methods | MSM, Trans people,female sex workers, and persons who inject drugs | 32 | General mental health | n/a | Worry about income was the most commonly reported issue among all groups of PLHIV. It was especially difcult for FSW because of COVID-19, as all restaurants, hotels, and hostels were closed, and their customers were also restricted from travelling. Accessing treatment proved to increase anxiety for PLHIV. Stigma and lack of disclosure of HIV status also seemed to contribute to psychological strain for some respondents during the quarantine period. | n/a | n/a |
| Wagner (2021) | Uganda | Mixed-Methods | Adult population Living with HIV | 280 | Depression | PHQ-8 | 6.1% had elevated depressive symptoms at baseline, and 17.9% after 12 months. Among those with elevated depressive symptoms post lock- down, the most common depressive symptoms (present at least most days in the past two weeks) were difculty sleep- ing (68.0%), depressed mood (60.0%), and feeling bad about oneself (54.0%), poor appetite (52.0%), and loss of interest in normally pleasurable things (50.0%). | In multiple regression analysis, higher food insecurity [adj. OR (95% CI) = 4.64 (2.16–9.96)] and perception that the pandemic negatively impacted ART adherence [adj. OR (95% CI) = 1.96 (1.22–3.16)] remained associated with a greater likelihood of elevated depressive symptoms | n/a |
| Wang (2021) | United States | Quantitative | PLWH users of marijuana | 222 | Anxiety, Depression, PTSD | GAD 7 PHQ-8 PC-PTSD | Cannabis use disorder: 9.7% moderate and 17% severe; Anxiety: 15.7% moderate and 13.2% severe; Depression: 16.1% moderante and 13.3% severe; PTSD: 30.4%. | Chi-square tests showed that participants were more likely to increase marijuana use during the COVID-19 pandemic if they reported worsened overall or mental health, perceived that marijuana use would not increase their risk for COVID-19 infection or severe symptoms. PTSD and a overall worse perceived mental health was associated with a changes in marijuana use. | n/a |
| Wang (2022) | Dominican Republic | Quantitative | Female sex workers | 187 | COVID-19–related Mental Health Challenges, Substance use, | the GAD-7, CES-D, and the Impact of Event Scale | The mean score was 7.06 (SD = 2.87) for the COVID- 19–related mental health challenges, indicating that on aver- age, the participant reported “1–2 days” in the past week for each question related to mental health challenge. The score was significantly higher for those reporting reduced HIV care vs. those who did not | increase in emotional partner abuse | n/a |
| Weerasuria (2021) | Australia | Quantitative | Adult population living with HIV | 153 | 86 (66%) reported worrying sometimes or often about their mental health. | n/a | not sure about this one - 86 (66%) reported worrying sometimes or often about their physical health and mental health, respectively, but not saying how they measured mental health. May exclude | n/a | n/a |
| West (2021) | Uganda | Qualitative | Adult population living with HIV and health workers | 10 | Distress, Worry, Fear | Participants were also asked to describe how COVID-19 impacted how people living with HIV experienced the mental health issues discussed earlier in the interview | Following the lifting of lockdown measures, most participants reported that transport costs were infated and that many people had lost their employment and could not aford the cost to travel to the clinic, which led to continued stress. Many participants discussed fear over the double burden stressor of getting COVID-19 while being HIV-positive and noted that this fear is particularly salient for people living with HIV. All participants said that lockdowns impacted people’s ability to work, throwing many into dire economic situations | our findings suggest this fear is even more prominent among people living with HIV.  Specifically, concerns over HIV disclosure are commonly associated with anxiety, fear and worry. From our data, COVID-19 curfew and travel restrictions placed people living with HIV who had not disclosed their status in situations where they had to newly contend with conceal- ing their HIV status, thus impacting their mental health by adding fear and anxiety over the possibility of inadvertent disclosure. | n/a |
| Wion (2021) | United States | Quantitative | n/a | 85 | depressive symptoms anxiety stress | Patient Reported Outcomes Measurement Information System (PROMIS) Item Bank v1.0—Emo- tional Distress—Depression—Short Form 8a instrument; Anxiety was measured using the PROMIS Item Bank v1.0—Emotional Distress—Anxiety—Short Form 8a instrument;  NIH Tool- box® Item Bank/Fixed Form v2.0—Perceived Stress (Ages 18 +) | Several participants reported being diagnosed with a chronic health condition with depression (36.5%, n = 31), anxiety (35.3%, n = 30), and hypertension (34.1%, n = 29) being the most frequent.  There was an increase in depressive symptoms from pre-pandemic (M=16.17, SD=6.34) to during the pandemic (M=18.17, SD=8.16) a difference of − 2.0 (95% CI, − 3.193, − 0.807), t(80) = − 3.34, p < 0.001), d = 0.37). Similarly, there was an increase in anxiety symp- toms from pre-pandemic (M = 17.20, SD = 6.92) to during the pandemic (M=20.20, SD=8.16), a difference of − 3.0 (95% CI, − 4.511, − 1.489), t(79)=− 3.95, p<0.001, d = 0.44.  There was an increase in stress from pre-pandemic (M=25.60, SD=6.03) to during the pandemic (M=27.52, SD=6.91), a difference of − 1.91 (95% CI, − 2.915, − 3.801) t(80) = − 3.80, p < 0.001, d = 0.42. | n/a | n/a |
